# Successful Repair of Concomitant Acute Type A Aortic Dissection and Saddle Pulmonary Embolism

**DOI:** 10.1055/s-0038-1639345

**Published:** 2018-07-27

**Authors:** Fabio Ramponi, Theone Papps, James Edwards

**Affiliations:** 1 Department of Cardiothoracic Surgery, Royal Adelaide Hospital, Adelaide, Australia

**Keywords:** aortic dissection, pulmonary embolism, concomitant

## Abstract

Patients presenting with acute onset of chest pain require prompt diagnosis and rapid establishment of a management plan. Acute aortic dissection and acute pulmonary embolism are life-threatening emergencies that can mimic each other at presentation. Correct identification of this uncommon scenario is crucial to initiate the appropriate interventions. The authors present a unique case of concomitant acute type A aortic dissection and acute saddle pulmonary embolism; the patient underwent successful aortic repair and pulmonary embolectomy.

## Introduction

Chest pain is a common presenting complaint accounting for millions of emergency department (ED) visits. Diseases of the heart, aorta, lungs, esophagus, stomach, mediastinum, pleura, and abdominal viscera may all cause chest discomfort. Clinicians in the ED focus on the immediate recognition and exclusion of life-threatening causes of chest pain such as acute coronary syndrome, acute aortic dissection (AAD), and pulmonary embolism (PE). A thorough medical history, with particular emphasis on the onset and quality of pain and associated symptoms, followed by a detailed physical examination is of central importance to organize appropriate investigations. Nevertheless, often these pathologies can be clinically difficult to differentiate especially when they coexist. AAD and acute saddle PE, although very rarely, can present simultaneously; we present a case where this unique high-risk scenario was promptly diagnosed and successfully managed with concomitant aortic repair and pulmonary embolectomy. To our knowledge, this has not been reported before.

## Case Presentation

**Fig. 1:**
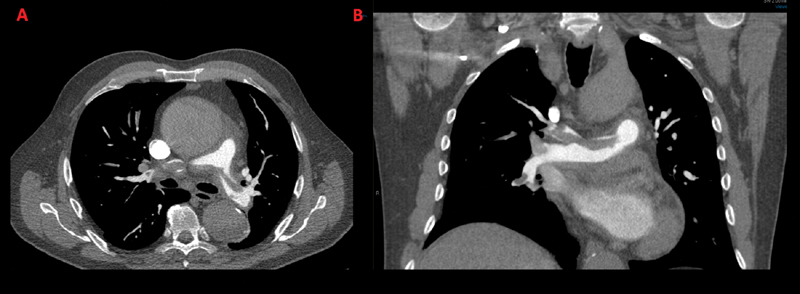
Computed tomography pulmonary angiogram showing large saddle pulmonary embolism in axial ( **A** ) and coronal ( **B** ) views. The ascending aorta is dilated and presents intraluminal abnormalities suspicious of an intimal flap.

**Fig. 2:**
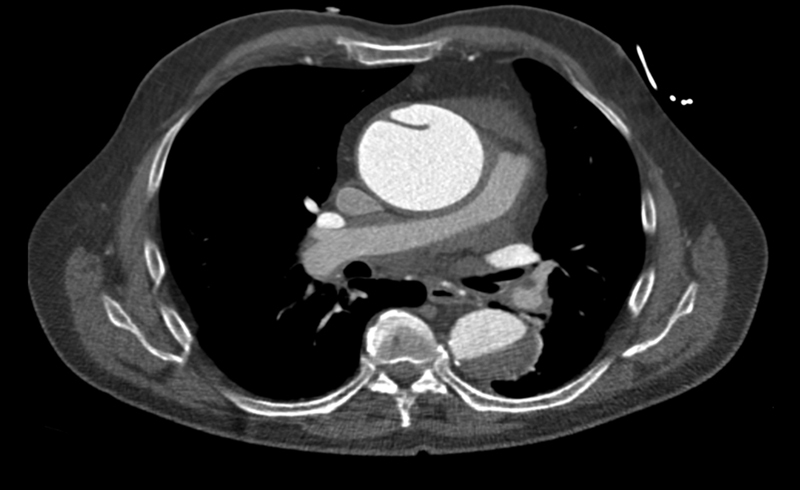
Computed tomography aortogram confirming the diagnosis of acute type A aortic dissection.

A 75-year-old man presented at our ED 2 hours after sudden onset of severe central chest pain that followed a straining effort; the pain was described as sharp, radiated between the scapulae, and was associated with palpitations and dyspnea. The only relevant past medical history was a spontaneous superficial thrombophlebitis a month prior; this was managed in the community without anticoagulation. The physical examination revealed an aortic regurgitation murmur and chest X-ray showed a moderately enlarged mediastinum. An urgent computed tomography (CT) pulmonary angiogram was organized ( Fig. 1 ); this showed (1) a large saddle pulmonary embolus extending into both left and right pulmonary arteries, (2) a dilated 7 cm ascending aorta with an apparent flap, and (3) a hemopericardium (Hounsfield Unit 30). Given the highly suspicious appearance of the ascending aorta, a CT angiogram was then performed confirming acute type A dissection. The dissection flap involved the ascending aorta and the aortic arch, sparing the supra-aortic vessels; a thrombosed false lumen extended into the descending thoracic and abdominal aorta just above the renal arteries ( Fig. 2 ). An urgent transthoracic echocardiogram confirmed the diagnosis and documented severe aortic regurgitation. At this stage, the patient was hemodynamically stable; decision was made to proceed immediately with aortic dissection repair and pulmonary embolectomy.

Femorofemoral bypass was established and at pericardiotomy a large hemopericardium was evacuated. The aorta was transected under deep hypothermic circulatory arrest (18°C) with retrograde cerebral perfusion. The entry tear was found in the anterior wall of the ascending aorta with fresh clot within the false lumen. The aorta appeared to be ruptured posteriorly with just a thin layer of clot tamponading against the main pulmonary artery bifurcation, preventing free intrapericardial rupture.

The ascending aorta was replaced with a 38 mm Dacron graft. After performing the distal anastomosis just proximal to the innominate artery, cardiopulmonary bypass was re-established and the main pulmonary artery was opened longitudinally. Embolectomy of a large saddle-shaped pulmonary thrombus (6 cm long) was then performed using Rampleys forceps. No attachment to the pulmonary artery intima was noted, confirming the acuteness of the embolic event.

Finally, the aortic root was replaced with a 29 mm Medtronic Freestyle stentless bioprosthesis (Medtronic). The patient was weaned off bypass uneventfully and transferred to the intensive care unit in stable condition.

The postoperative course was unremarkable. No deep vein thrombosis or thrombophilic traits were identified. Pathology of the pulmonary embolus showed characteristic lines of Zahn, indicating a recent event. The patient was discharged home on lifelong warfarin on postoperative day 10.

## Discussion

Acute aortic dissection accounts for 85 to 95% of all acute aortic syndromes and its incidence is ∼15 cases per 100,000 patient-years. Chest or back pain, often described as “sharp,” is the most frequent presenting symptom (84.8%). Hypotension, pulse deficit, and acute heart failure are the most common physical findings. 1 In the general population, the incidence of PE has increased to 112 cases per 100,000 patient-years and patients usually present with dyspnea and pleuritic chest pain. Saddle embolus accounts for 3 to 6% of PE cases. Those patients are more likely to experience hemodynamic instability and their acute mortality is 5%. 2

AAD and PE can mimic each other clinically. Rapid differential diagnosis is critical to establish the correct treatment and improve outcome. D-dimer is a biomarker with good sensitivity (93.5%) but low specificity (54%) for AAD. 3 In the diagnosis of PE, sensitive D-dimer testing is mostly useful in conjunction with clinical suspicion to guide further investigations. Triple-rule out (TRO) CT angiography to simultaneously evaluate acute coronary syndrome, acute aortic syndrome, and PE is increasingly being performed in institutions where a 64-slice multidetector CT scanner is available. Despite its valuable role, TRO carries a higher radiation exposure due to the extended z-axis coverage. For these reasons, Lee et al suggested that the use of dedicated coronary CT angiography (DTCA), instead of a TRO protocol, is equally safe when triaging patients with nonspecific acute chest pain. 4 Pulmonary transit time needs to be taken into account when performing DTCA in this scenario to avoid a false-negative diagnosis of PE. 5

Transesophageal echocardiography is comparable to a CT angiogram for sensitivity and specificity in the diagnosis of AAD. 1 However, echocardiography cannot definitively diagnose PE. In stable patients, it may be used when other tests are inconclusive and clinical suspicion remains high. About 30 to 40% of patients with massive PE have echo signs of right ventricle (RV) strain or overload, like RV enlargement, RV dysfunction, or tricuspid regurgitation. However, in case of AAD with intrapericardial posterolateral rupture, the compression onto the main pulmonary artery can result in acute RV dilatation and dysfunction, resembling the appearance of a massive PE. 5

Although differential diagnosis is crucial, physicians should bear in mind that on rare occasions aortic dissection and PE can coexist. This has important implications in the decision making and management plans. Type A dissection with associated embolism of the right main pulmonary artery was reported by Leu and Yu; the patient refused surgery and died few months after. 6 More recently, another case of acute type A dissection limited to the ascending aorta and concomitant lobar PE was reported by Tudoran and Tudoran; the patient underwent successful aortic surgery 1 month after the diagnosis. 7 Finally, peripheral PE associated with acute type A dissection was described by Herrera et al in a patient with Marfan syndrome and hyperhomocysteinemia, who survived aortic surgery. 8

In our case, the patient presented with sudden onset of sharp chest pain without other associated symptoms. The history of recent thrombophlebitis led the ED physician to investigate with a CT pulmonary angiogram that revealed a large saddle PE. The observation by an alert radiologist of a dilated and abnormal ascending aorta prompted a completion CT aortogram, confirming the suspicion of simultaneous type A dissection. Correct diagnosis of concomitant pathology was crucial; establishment of therapeutic anticoagulation, in fact, would have carried devastating consequences in the setting of intrapericardial rupture of the dissection.

In conclusion, AAD and saddle PE, although very rarely, can present simultaneously. Emergency clinicians must guard against premature diagnostic closure when assessing patients presenting with acute chest pain.
